# Minimally invasive surgery in older children with Hirschsprung’s disease in a North African Country

**DOI:** 10.3389/fsurg.2022.934289

**Published:** 2022-12-22

**Authors:** Ahmed Arafa, Moutaz Ragab, Osama Abdelazim, Sayed Khedr, Wesam Mohamed

**Affiliations:** ^1^ Department of Surgery, Pediatric Surgery Unit, Cairo University Specialized Pediatric Hospital (CUSPH), Cairo, Egypt; ^2^ Faculty of Medicine, Cairo University, Cairo, Egypt

**Keywords:** Hirschsprung, minimally invasive surgery, Swenson pull-through, Duhamel, laparoscopy

## Abstract

**Introduction:**

Hirschsprung's disease (HSD) is a bowel congenital anomaly affecting mainly the enteric nervous system of the rectosigmoid region. Surgical resection of the aganglionic segment and restoration of bowel continuity *via* coloanal anastomosis is the main stay of treatment. In 1999, Georgeson et al. introduced a new minimally invasive approach as a standard for the pull-through mechanism. This study aims to evaluate the safety and possibility of the use of a laparoscope in older children with HSD with various techniques for HSD surgery.

**Methods:**

This study was performed based on 20 patients diagnosed with HSD. The patients are older children, whose mean age is 3 years. The cases showing enterocolitis or obstruction were excluded from the study. We divided these cases into two groups: Group A, consisting of 10 cases where laparoscopic-aided transanal pull-through was done, and group B, in which the laparoscopic Duhamel procedure was done.

**Results:**

We compared between two groups for the first year follow-up period. In Group A, there were two cases of stenosis that respond to regular dilation: one case of enterocolitis and one case of fecal incontinence. In Group B, we had two cases of constipation and three cases of enterocolitis. There was no anastomotic leak in both groups.

**Conclusion:**

Minimally invasive surgery is safe in management of HSD in older children in one stage, either by using the Duhamel or transanal Swenson procedure.

## Introduction

Hirschsprung's disease (HSD) is a bowel congenital anomaly affecting mainly the enteric nervous system in the rectosigmoid region in which there is absence of the parasympathetic ganglions in the intestinal submucous and myenteric plexuses (1, 2).

Surgical resection of the aganglionic segment and restoration of bowel continuity is the mainstay for treatment of HSD. Over the past few decades, many surgical procedures were developed by surgeons such as Swenson, Duhamel, and Soave to correct this anomaly (3). Transanal pull-through for HSD is a safe procedure for both neonates and infants. However, overstretching the anal sphincter and sigmoid colon mesentery might have the potential risk of impaired defecation function (4).

In 1999, Georgeson et al. introduced a minimally invasive technique for management of HSD as a new standard. Gradually, it gained popularity in many centers concerned with managing HSD (1). Leveling biopsies, bowel mobilization under vision, and cosmetic superiority are added values to the laparoscopic approach.

Duhamel’s procedure is widely used as it is technically easy, requiring less anal stretching, especially in older children. Travassos et al. reported that the Duhamel procedure is the best one for a “failed” Swenson operation, long-segment HSD, and total colonic aganglionosis, and in cases of difficult mucosectomy, repetitive attack of enterocolitis makes the dissection difficult and hypertrophic rectum grossly dilated (5).

The current study is evaluating the outcome of minimally invasive surgery techniques for treating children older than 3 years presenting with HSD from surgical and functional perspectives.

## Materials and methods

This study has been conducted based on 20 patients diagnosed with HSD. They are older children whose mean age is 3 years. They were brought to our hospital between March 2018 and March 2021. We excluded the cases who were less than 1-year-old infants, showing enterocolitis or obstruction; also, we excluded cases that needed ileostomy that did not respond to rectal irrigation by barium enema, which was performed to reassess colonic dilation and was also proved by a laparoscope. We divided these cases into two groups: group A comprises 10 cases for whom laparoscopic-aided transanal pull-through was done and Group B comprised of those in whom laparoscopic Duhamel procedure was done.

We have followed-up these cases 1 year postoperatively. We informed their parents and obtained their consent; diagnosis of HSD was recorded in the form of history of delayed passage of meconium, history of recurrent constipation, usage of suppositories, enemas, clinical examination in the form of abdominal distention, and associated anomalies; general examination was also done to exclude obstruction or enterocolitis. Investigations were conducted in the form of barium enema to determine the funnel sites, and rectal biopsy revealed the absence of ganglia using hematoxylin–eosin staining.

Then, all patients underwent mechanical and chemical preparation: mechanical preparation was in the form of rectal irrigation using saline for 2 months 20 ml/kg daily to decompress hugely dilated colon, to do resection and pull-through without colostomy as one stage, as one operation, and to facilitate anastomosis, minimize incidence of leakage, and fecal incontinence. The chemical preparation was in the form of prophylactic preoperative antibiotics to cover gram-positive, gram-negative bacteria and anaerobes.

### Operative procedure

Patients were placed in the Trendelenburg position in cases of rectosigmoid HSD. This position was changed into the reversed Trendelenburg position in case of the long segment when the transitional zone was proximal to the transverse colon. The pressure was maintained at 10–12 mm Hg, flow at 3/min. Three ports were inserted, and a camera port was also inserted at the umbilicus. The other two ports inserted were as follows: one was inserted below the xiphisternum, and the other was inserted at the right lower quadrant. In only three cases with the long segment, we needed an additional port in the left lower quadrant.

There were two parts: a laparoscopic part and a transanal part.

**First: laparoscopic part:**

Laparoscopic colonic biopsies were taken for a fresh frozen section to determine the level of resection at ganglionated pull-through in an ascending manner especially in cases with an ill-defined funnel.

**Figure 1:**
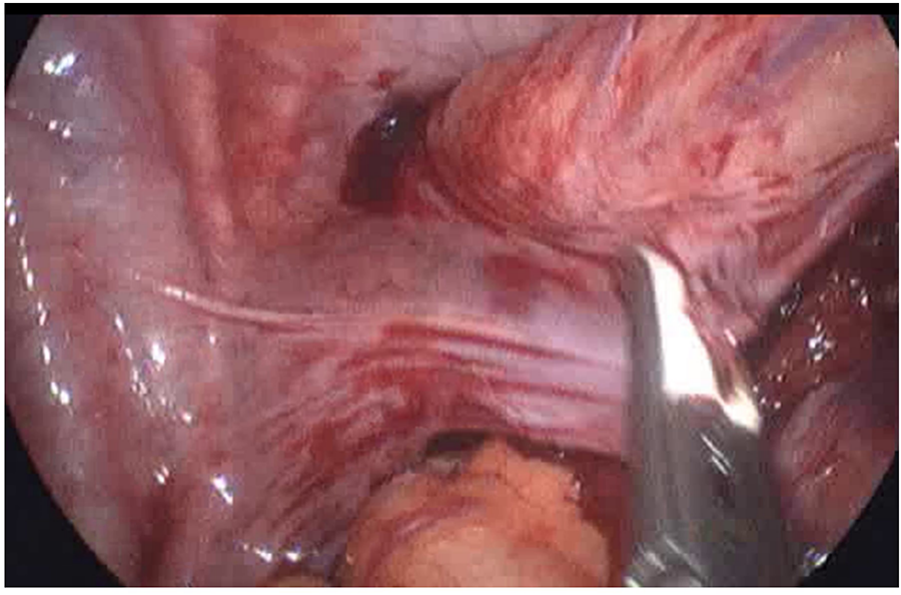
Devascularization of the distal part.

**Figure 2:**
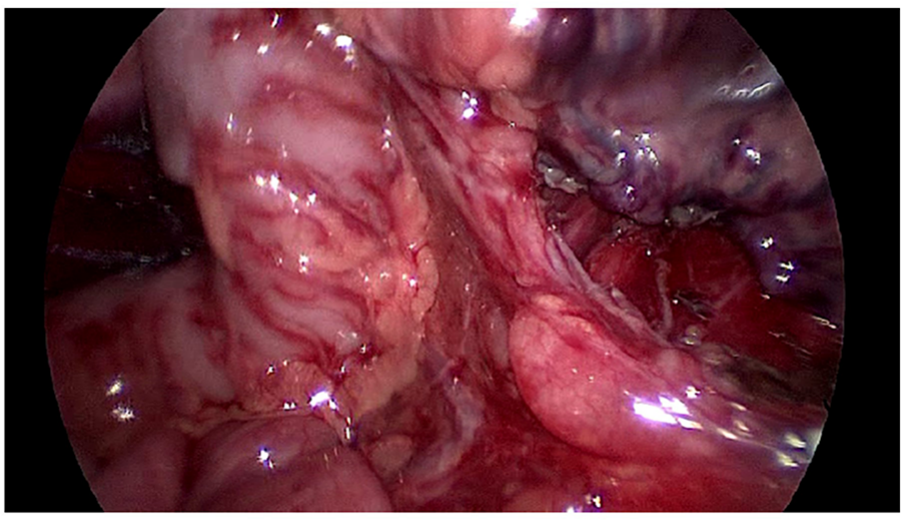
Mobilization of the proximal part.

The rule of operation was mobilization in the proximal part to preserve vascularity of the pulled colon and devascularization in distal part (Figures 1, 2).

In long segments, division of the splenic flexure and division of the lower left colic vessels were implemented.

**Figure 3:**
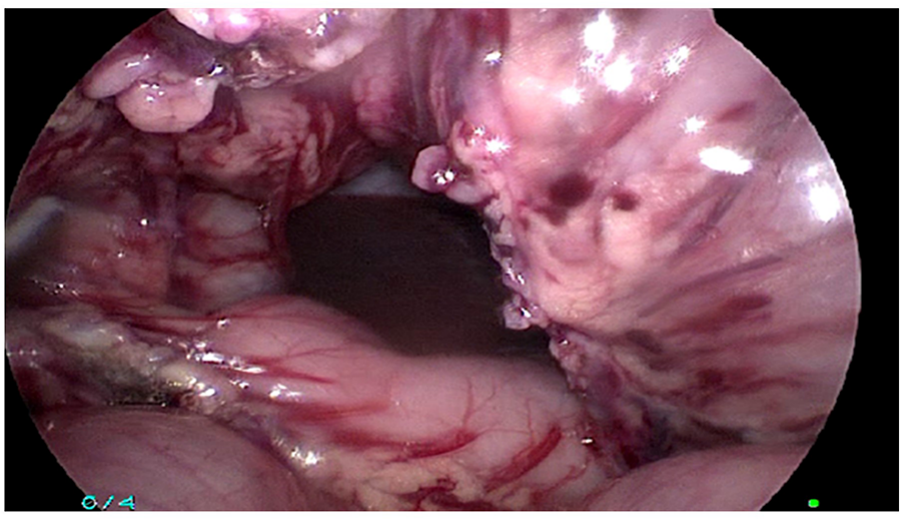
The transverse colon was dissected from the gastrocolic ligament; upper left colic vessels and middle colic vessels were divided preserving a marginal arcade.

If the transitional zone was proximal to the transverse colon, the transverse colon was dissected from the gastrocolic ligament; upper left colic vessels and middle colic vessels were divided preserving marginal arcade (Figure 3).

**Figure 4:**
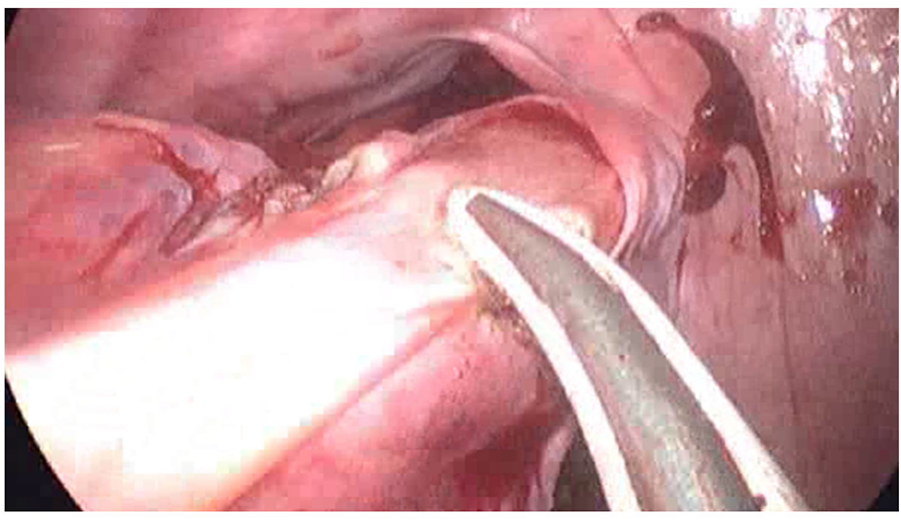
Group A: all circumference of the aganglionic rectum was mobilized.

**Figure 5:**
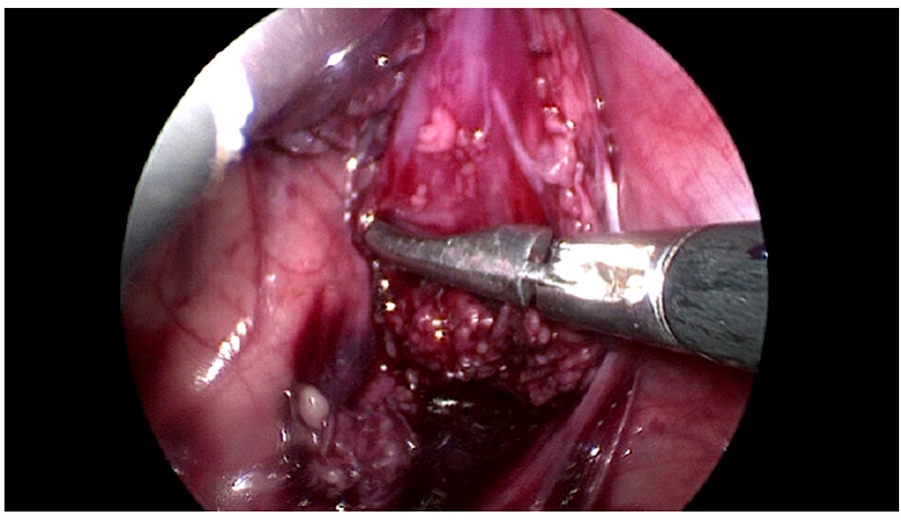
Group B: only the posterior part of the rectum was mobilized in a retrorectal avascular plane.

**Figure 6:**
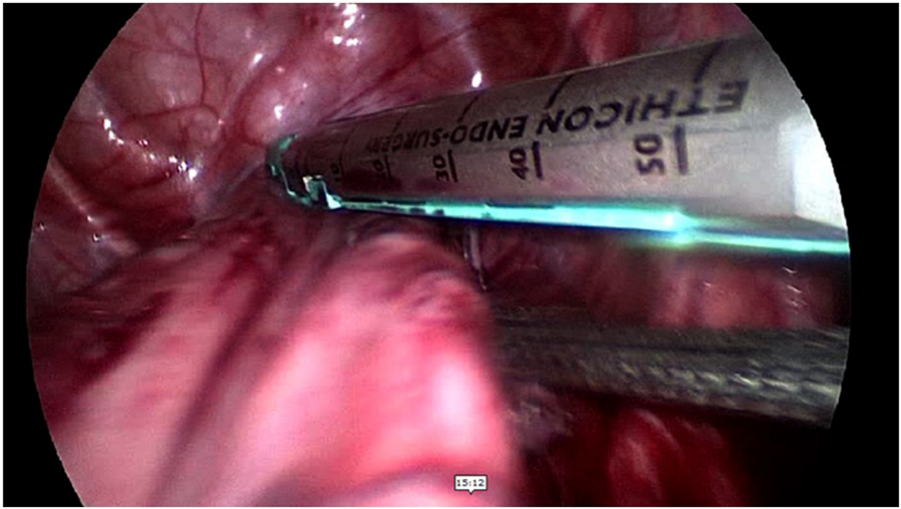
A gastrointestinal stapler was used to divide the aganglionic rectum as low as possible.

In both groups, incision of peritoneal reflection was done; in Group A, all circumference of aganglionic rectum was mobilized, while in group B, only the posterior part of the rectum was mobilized in a retrorectal avascular plane (Figures 4–6) guided by rectal examination 1 cm above the dentate line. In both groups, dissection was done below peritoneal reflection 2–3 cm as much as possible.

**Figure 7:**
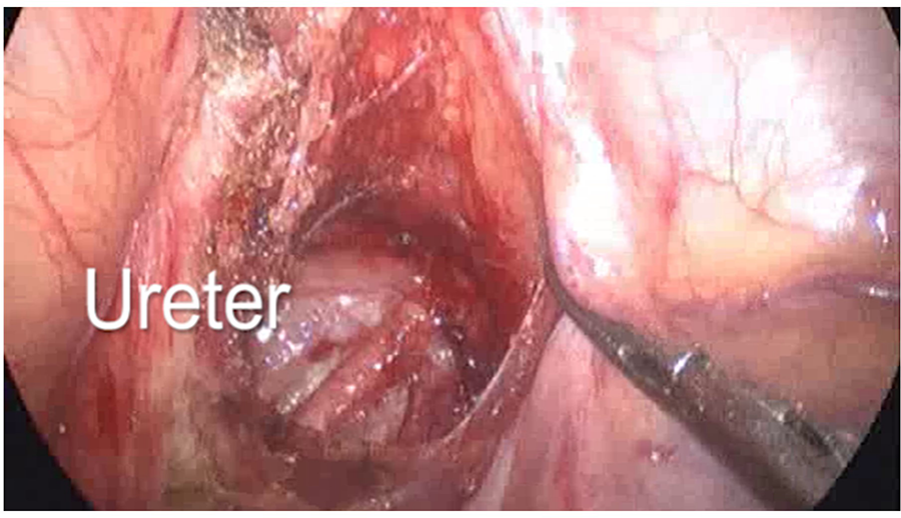
Clear identification of the pelvic structures.

In group B, a 55 mm linear cutter gastrointestinal stapler was used to divide the aganglionic rectum as low as possible to leave only a short aganglionic rectal stump (Figure 7).

**Second: transanal part:**

In group A, a circular incision in the rectum 1 cm above the dentate line was carried out, and then rectal dissection for a distance 2–3 cm only as most of the rectum was laparoscopically dissected; the plane of dissection was very close to the rectal wall, and then we pulled the dissected rectum, which was resected, and anastomosis was done.

In Group B, a lower semicircular incision was done in the posterior rectal wall 1 cm above the dentate line; multiple stay sutures were taken in the incised posterior rectal wall to help in doing anastomosis with the pulled through neocolon. Anastomosis between anterior neocolon and the held posterior rectal wall was done by Vicryl 3/0, and for anastomosis of the rest of neocolon with the anal mucosa above the dentate line from 3 to 9 o’clock, a linear GI STAPLER 75 was used to make common wall between posterior rectal wall and anterior wall of the neocolon.

Laparoscopically, we checked to detect that there was no intra-abdominal bleeding and no colon twisting.

## Results

Twenty patients, who are older than 3 years, presented to our hospital, 13 males and 7 females. The age of the patients ranged from 3 to 8 years. In both groups, we started oral intake after 48 h postoperatively.

Table 1 summarizes the results of our patients.

**Table 1:** Patients data and results.

	Group A	Group B
Operative time in rectosigmoid HSD (h)	2–3	3–4
Operative time in long-segment HSD (h)	3–4	4–5
Leakage	No	No
Perianal excoriation	Three cases	Two cases
Enterocolitis	One case	Three cases
Hospital stays (days)	4–5	4–5
Constipation	One case	Two cases
Regular dilation	Two cases needed regular dilation	No
Anal Stenosis	Two cases	No
Spur formation	No	One case
Fecal incontinence	One case	No

HSD, Hirschsprung's disease.

## Discussion

Georgeson et al. (1) reported the use of a laparoscope in HSD disease (1), while De La Torre and Ortega and Langer et al. (2, 3) introduced complete transanal endorectal pull in HSD. Visser et al. (4) preferred both techniques than the open technique, while Kohno et al. (6) reported that the transanal endorectal pull-through (TERPT) with or without the usage of laparoscope for HSD has been widely applied.

**Figure 8:**
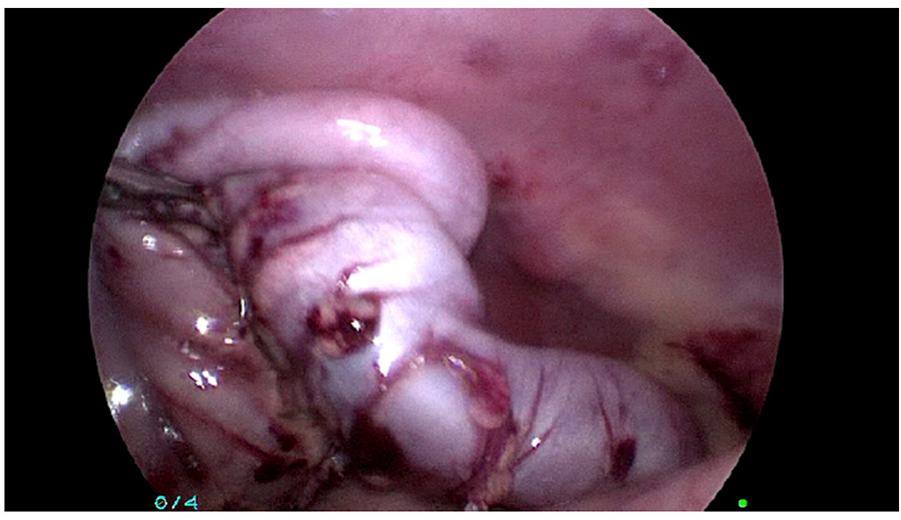
Transitional zone identification.

Laparoscopy in cases of Hirschsprung’s disease helps in clear identification of pelvic structures (Figure 7), less postoperative time, better cosmetic results, and also to identify the transitional zone (Figure 8) (7–9). It is also superior to the pure transanal technique in many aspects in the operation, e.g., avoiding twisting or tension of the pull-through segment (10–12).

Yehya et al. (13) had 40 cases, median age of 2 years, divided into two groups: group A was the laparoscopic-aided transanal endorectal pull-through, and group B was the pure transanal endorectal procedure, but Keckler et al. (14) preferred to do either the Soave or Swenson technique aided by a laparoscope. In our study, we divided laparoscopic-aided transanal pull-through and laparoscopic Duhamel procedure into two groups.

Hadidi (15) and Langer et al. (16) reported that the role of laparoscopy is limited to those with the long aganglionic segment, whereas in our study, we used laparoscopy not only in the long segment but also in the short segment owing to several advantages, e.g., preservation of anal sphincter by minimizing excessive manipulation and rectal dissection transanally, which occurred in pure transanal rectal pull-through.

Langer et al. (16) reported that clinical presentations of their cases range from infants’ bowel obstruction to progressive chronic constipation at an older age; also, in this study, main manifestation in our cases is chronic constipation.

Takawira et al. (17) reported that they depend on intraoperative fresh frozen sections to confirm diagnosis and to determine the limit of resection by detection of the ganglionic part to be pulled through after resection of the aganglionic part, but in this study, intraoperative laparoscopic biopsies for fresh frozen sections were done in cases of ill-defined funnel only.

Tang et al. (18) had 182 patients with Hirschsprung’s disease with different age groups: laparoscopic-assisted endorectal Soave pull-through procedure for their cases was done and two cases of intestinal herniation from the trocar site was done; 32 cases complained of perianal excoriation, and anastomotic leakage occurred in three cases; also, postoperative adhesive bowel obstruction was detected in 2 cases (1.1%), 14 cases had enterocolitis (7.7%), 4 cases had anastomotic stenosis (2.2%), recurrent constipation occurred in 3 cases (1.6%), and 7 cases had soiling (3.8%). Gadallah et al. (19) reported that postoperative fecal incontinence may be due to the intraoperative sphincter damage, while Yehya et al. (13) reported that after 1 year of follow-up, continence score was normal in 10 (50%), good in 9 (45%), and fair in 1 (5%) in the laparoscopic-assisted transanal endorectal pull-through group, while the continence score was normal in 5 (25%), good in 14 (70%), and fair in 1 (5%) in the pure transanal endorectal pull-through group. Improvement in stooling score in the laparoscopic group could be explained by better sphincter function due to less sphincter damage, with the avoidance of overstretching the anal sphincters in the pure trans anal pull through procedure. In this study, in group A, there were two cases of stenosis that responded to regular dilation; one case of enterocolitis postoperatively that responded to conservative treatment only in the form of parenteral antibiotics, rectal irrigation, intravenous fluids, and NP0; and one case of fecal incontinence. In Group B, we had two cases of constipation and three cases of enterocolitis, There was no anastomotic leak in both Groups.

## Conclusion

Minimally invasive surgery is safe in managing HSD in older children in one stage either through the using Duhamel procedure or the transanal Swenson procedure.

## Data Availability

The raw data supporting the conclusions of this article will be made available by the authors, without undue reservation.
